# Surgery of the Lungs and Pleuræ

**Published:** 1894-05-12

**Authors:** 


### SURGERY OF THE LUNGS AND PLEURÆ.

### Empyema.

The surgical treatment of pleurisy and empyema in children is discussed by Mr. Godlee in the "International Clinics."[1] He has little to say about the treatment of serous effusion, but his remarks on empyema are of much value. Recognising the fact that very occasionally aspiration has been successful (though seldom after one operation), he thinks it does not save time, and that the sooner a free opening is made into the chest the better. This should, he considers, be done in all cases with, perhaps, the following exceptions: If associated with phthisis, unless to relieve cardiac or respiratory difficulty, because he does not think a cure likely to result, but, on the other hand, the tuberculous process would be accelerated. If the empyema has already opened through the lung, he advises us to wait a short time and see if it clears up. He does not consider the spontaneous evacuation of an empyema satisfactory, and advises operative interference in all such cases. If the empyema has already opened into a bronchus, the danger of deep anæsthesia, allowing the entrance of pus into the air tubes, which cannot be expectorated, is referred to. The best place for opening the empyema is discussed, and the preference is given to a spot opposite the ninth rib and a little outside the line of the angle of the scapula. The objection raised to the sixth interspace in the axillary line is that very often a pocket of pus may remain at the back of the chest; but on the other hand we must not forget that if the patient lies on the affected side, as he commonly does, the lowest point for drainage is the axilla and not the base behind, and that if we drain behind the diaphragm rising up last in the axilla may leave a pocket of pus there. In a child, Godlee always excises a portion off the rib to get a large enough opening. Perhaps this might not be so necessary in the wider axillary interspaces if the opening were made there. The rib is reproduced in two months. The tube should be only just long enough to enter the cavity; a long tube lying in it only irritates.

The difficulty in getting some cases of empyema which have opened into the lung to heal, even after a free incision and drainage of the pleura, is alluded to, and it is recommended that the external opening should be maintained for a long time.

The question of removal of portions of the chest wall to allow of obliteration of empyemata is referred to, and we are told that where the chest wall has fallen in as far as it can and the lung expanded to the utmost extent, and yet the cavity is not obliterated, we should perform this operation. But the difficulty lies in deciding when the chest wall and lung have done their part to the utmost, and probably we must rather be guided by the direction of the empyema, but it is generally considered that Estlander's operation is seldom required in children.

Dr. Sutherland[2] discusses the mechanism by which obliteration of the pleural cavity takes place in the evacuation of an empyema. He believes that the chief factors in producing this result are two in number, (1) the re-expansion of the lung, and (2), this forcible elevation of the diaphragm. The re-expansion of the lung is, he believes, mainly due to expiratory efforts with the closed glottis as in coughing, by which means air is driven into the collapsed lung. The elevation of the diaphragm is brought about by the contraction of the muscles in the anterior abdominal wall.

Dr. Sutherland advocates very early removal of the tube, by the third day in most cases. He thinks if the external opening closes, and air is therefore shut out, the absorbing power of the pleura will soon remove any pus remaining in the cavity. We should, however, certainly rather trust to drainage, avoiding of course the projection of the tube into the cavity, so as to act as an irritant to the pleura.

An interesting case of empyema occurring in a patient with phthisis, and discharging through a bronchus, is reported by Dr. Alexander James.[3] The presence of tubercle was alone diagnosed by finding the tubercle bacilli in the sputum, as the fluid in the pleura obscured the signs of tuberculosis in the lung. Dr. James thinks it is safest in these cases not to aspirate, but to open the pleural cavity by means of a free incision, so as not to allow the collapsed lung to expand rapidly, a condition which in tuberculous disease of the lung he has known to be followed by pneumothorax in one case, and severe hæmoptysis in another.

Dr. Achütz,[4] in a paper read before the Medical Society of Hamburgh, advocates resection of a portion of rib in opening an empyema in children, and that the opening should be made far back, near the vertebral column. He leaves the drianage tube in for three weeks. We are surprised to see that he advises washing out, which in this country we regard as dangerous, and only to be performed in cases where the discharge is offensive. We notice that Aufrecht,[5] of Magdeburgh, also advocates washing out with 2 per cent. carbolic solution. Gaston,[6] on the other hand, believes it is only requisite when the fluid is septic. It is proposed by Dilorme[7] to treat cases of chronic empyema by peeling off the thickened pleura from the lung, which, he says, admits of its expansion.

A case in which puncture of a pneumothorax, due to perforation by a valvular aperture of a tubercular cavity, was followed by very extensive subcutaneous emphysema, which threatened to asphyxiate the patient, is recorded by Dr. Galliard[8] as a substitute for Estlander's operation. Dr. Jaboulay[9] suggests desternalisation of the ribs, by which he means division of the costal cartilages. He finds on the dead body that the weight of the arm drags the first and second ribs away from the sternum, but the other ribs tend to overlap it, and thus contract the chest on that side. He thinks this general contraction will do more to obliterate the cavity in old-standing cases of empyema than resection of portions of a few ribs.

A most remarkable treatment of chronic empyema is recorded by Dr. Fowler, of Brooklyn.[10] A large cicatricial mass through which the sinus ran was dissected out of the interior of the chest, and away from the pericardium. The operation seems to have been of the most dangerous character, but the result was very satisfactory, as the cavity closed in a month. The sinus was found to be lined with epithelium, and contained many rudimentary sebaceous glands.

The resection of a growth from the chest wall, which involved the surface of the lung, has been recorded by Müler.[11] He prevented lung collapse by "lifting the tumour and lung into their normal relations." The patient recovered. He believes these malignant growths of the chest wall involving the lung can be successfully removed in other cases.

*Injuries of the Chest.*—It seems hardly possible that a person should survive the opening of both pleural cavities at the same time, and yet such a case is recorded by Mr. Bilton Pollard[12]. A boy fell a great height on to some railings, and both sides of the chest were lacerated, opening both pleuræ. The boy's condition was, of course, an exceedingly grave one, but by bringing the edges of the wounds into accurate apposition, during expulsion of the air from the pleura with expiration, the boy recovered, a moderate serous effusion taking place on one side. Mr. Bartlett[13] suggests that in these cases we should inflate the lungs, as we do in asphyxia neonatorum by blowing through a tube held firmly between the patient's lips, the nostrils being closed. This would, he thinks, expand them satisfactorily, driving the air out of the pleura. He has never had an opportunity of trying it on the living, but has shown that it is feasible on the cadaver. He also alludes to the method of removing the air from the pleura after the closure of the wound, by aspiration, as in a non-traumatic pneumothorax.

[1] International Clinics, vol. iv., 1893. [2] Lancet, Jan. 27, 1894, p. 198. [3] Edin. Med. Journal, Feb., 1894, p. 712. [4] Med. Record, Feb., 1894, p. 247. [5] Internat. Med. Magazine, Jan., 1894, p. 1151. [6] Journal American Medical Association, vol. xxi., No. 9, and Theraput. Gaz., Jan., 1894. [7] Epitome B. Med. Journal, Feb. 10, 1894, p. 22. [8] Medical Week, March 9, 1894, p. 116. [9] New York Med. Record, Feb. 17, 1894, p. 207. [10] Medical Record, Dec. 30, 1893, p. 838. [11] American Journal, March, 1894, p. 339. [12] Lancet, Feb. 17, 1894, p. 402. [13] Lancet, March 10, 1894, p. 641.

